# Making food labels social: The impact of colour of nutritional labels and injunctive norms on perceptions and choice of snack foods^☆^

**DOI:** 10.1016/j.appet.2015.03.034

**Published:** 2015-08-01

**Authors:** Milica Vasiljevic, Rachel Pechey, Theresa M. Marteau

**Affiliations:** Behaviour and Health Research Unit, University of Cambridge, UK

**Keywords:** Nutritional labelling, Colour labelling, Emoticons, Injunctive norms

## Abstract

Recent studies report that using green labels to denote healthier foods, and red to denote less healthy foods increases consumption of green- and decreases consumption of red-labelled foods. Other symbols (*e.g.* emoticons conveying normative approval and disapproval) could also be used to signal the healthiness and/or acceptability of consuming such products. The present study tested the combined effects of using emoticons and colours on labels amongst a nationally representative sample of the UK population (*n* = 955). In a 3 (emoticon expression: smiling vs. frowning vs. no emoticon) × 3 (colour label: green vs. red vs. white) ×2 (food option: chocolate bar vs. cereal bar) between-subjects experiment, participants rated the level of desirability, healthiness, tastiness, and calorific content of a snack bar they had been randomised to view. At the end they were further randomised to view one of nine possible combinations of colour and emoticon labels and asked to choose between a chocolate and a cereal bar. Regardless of label, participants rated the chocolate as tastier and more desirable when compared to the cereal bar, and the cereal bar as healthier than the chocolate bar. A series of interactions revealed that a frowning emoticon on a white background decreased perceptions of healthiness and tastiness of the cereal bar, but not the chocolate bar. In the explicit choice task selection was unaffected by label. Overall nutritional labels had limited effects on perceptions and no effects on choice of snack foods. Emoticon labels yielded stronger effects on perceptions of taste and healthiness of snacks than colour labels. Frowning emoticons may be more potent than smiling emoticons at influencing the perceived healthiness and tastiness of foods carrying health halos.

## Highlights

• Red and green labels have no significant effect on snack perceptions and choice.
• Emoticon labels implying injunctive norms affect perceptions of health and taste.
• Frowning emoticons may be more potent than smiling emoticons for certain foods.

## Introduction

One of the most pressing public health threats is the global obesity epidemic. Obesity, or body adiposity, is one of the four behavioural risk factors most strongly related to the steady rise of non-communicable diseases, including cardiovascular diseases, cancers, chronic pulmonary diseases, and diabetes (WHO, 2011). To tackle the global burden of obesity that disproportionately affects people from lower socioeconomic groups living in underdeveloped or developing countries (Di Cesare et al., 2013) we need innovative population-level interventions aimed at changing the “obesogenic environments” that have contributed to the obesity epidemic. One such population-level intervention is nutritional labelling on food products.

### Nutritional labelling

Several studies to date have looked at the effects of nutritional labelling, defined as information being given about at least one nutrient in either relative or absolute terms, where the information is visible at the point of purchase and consumption choice (see Crockett, Hollands, Jebb, & Marteau, 2011). Reviews of the evidence on the use of nutritional labelling demonstrate that reported use of nutritional labels by consumers is higher than actual use, and there are wide ranging differences in preferences of labelling format between consumers depending on the choice context (Cowburn & Stockley, 2005; Grunert & Wills, 2007; Malam et al., 2009). Whilst the number of high quality studies investigating how consumers use nutritional labels is limited, a recent synthesis of the evidence on actual purchasing and consumption found no significant effects of nutritional labelling (Crockett, King, Hollands, Jebb, & Marteau, under review; see also recent review by Hieke & Wills, 2012). One reason for these largely null findings could be the diverse range of nutritional labelling formats that vary significantly between countries, but also within countries including Traffic Light labels, Guideline Daily Amounts (GDA), Choices logos, and Facts up Front.

One frequently used labelling format has been colour labels, in particular traffic light colours (green, red, and amber), to provide nutritional information of foods. A recent lab study by Temple et al. (2011) found that using green labels to denote healthier foods and red to denote unhealthier options helped decrease consumption of red-labelled food options and increase consumption of green-labelled foods. Similar findings were obtained in a cafeteria setting by Levy, Riis, Sonnenberg, Barraclough, and Thorndike (2012), who found that labelling unhealthy products with a red label, and healthy products with a green label, led to healthier purchases of both beverages and food post-baseline.

However, other studies have found no evidence that colour labels increase perceptions of healthiness and greater consumption of healthier products. Recent studies in supermarket settings in the UK and Australia found that the introduction of front-of-pack traffic light labels did not lead to healthier food purchasing (Sacks, Rayner, & Swinburn, 2009; Sacks, Tikellis, Millar, & Swinburn, 2011). More recent research has established moderators of the effect of colour labelling. For example, traffic light labels were found to be only effective for people with low self-reported self-control (Koenigstorfer, Groeppel-Klein, & Kamm, 2014); and only when participants were asked to choose a product according to its perceived healthiness rather than their personal preferences (Aschemann-Witzel et al., 2013). Furthermore, the study by Aschemann-Witzel et al. only found the effects of colour labelling in German participants, but no such effects in participants in Poland. Thus, studies to date have not produced conclusive results as to the effectiveness of colour nutritional labels at improving the healthiness of food choices.

A recent paper by Schuldt (2013) tested the automatic influences of colour of nutritional labels on people's perceptions of food healthiness. Schuldt tested the hypothesis that a green nutritional label promotes inferences of a healthier product, compared to an identical nutritional label coloured in red. This hypothesis arises from embodied cognition whereby the colour green often implies safety and constitutes a ‘go’ signal in many cultures, whereas red confers danger and signals ‘stop’ responses, as evidenced in traffic signs. Consistent with this reasoning, Schuldt (2013) found that participants were more likely to perceive the same chocolate bar as healthier when the nutritional label was presented in green, as opposed to red (Study 1) or white (Study 2), despite the fact that the calorie information was held constant across conditions.

### Injunctive norms

In a related vein colour labels may be combined with other symbols that convey the social norms attached to a particular food product, and that could signal the healthiness and/or perceived acceptability of consuming such products. Social norms can be defined as implicit or explicit rules regarding appropriate behaviour within a particular social context. Reminding people that a deleterious behaviour is less prevalent in the population than assumed (a *descriptive norm*) leads to decreased engagement with the negative behaviour. However, such interventions often produce unintended “boomerang” effects, whereby people who already abstain from the undesirable behaviour actually inadvertently start engaging more with the negative behaviours in order to match the social norms surrounding that behaviour (Cialdini & Goldstein, 2004; Reno, Cialdini, & Kallgren, 1993). One way of ameliorating this “boomerang” effect may be to provide an *injunctive norm* that signals approval to the people who already disengage from the negative behaviour that the interventions aim to target.

This suggestion was tested in a recent study by Schultz, Nolan, Cialdini, Goldstein, and Griskevicius (2007) that examined the viability of using descriptive and injunctive norms to promote household energy conservation. Results demonstrated that high-energy-consuming households receiving only descriptive normative information reduced their energy consumption, whilst low-energy consuming households receiving only the descriptive message showed a “boomerang” effect, whereby they increased their energy consumption post-baseline. Adding an injunctive normative message (either approving “” or disapproving “” emoticons) to the descriptive normative message buffered successfully against this “boomerang” effect.

In the domain of food a recent systematic review by Robinson, Thomas, Aveyard, and Higgs (2014), demonstrated that descriptive norms influence food selection and amount of food eaten. Whilst the effectiveness of descriptive norms to improve people's food behaviours has been established, there has not been much research into the effectiveness of injunctive norms. To our knowledge only one study manipulated descriptive and injunctive norms for food consumption (Robinson, Fleming, & Higgs, 2014), finding that descriptive norms exerted influence on fruit and vegetable consumption, but no such effect was found for injunctive norms. The effectiveness of injunctive norms for food-related behaviours thus requires more systematic examination.

### The present research

There are several gaps in Schuldt's (2013) research reviewed above, which need to be addressed to understand how colour nutritional labels can promote healthier food choices. First, people inferred greater healthiness of a chocolate bar when seeing a green label as compared to a red label, but Schuldt's experiment did not show whether green labels increased perceptions of healthiness, or red labels decreased perceptions of healthiness, or indeed both. Second, Schuldt's experiments only tested the effects of colour nutritional labels for an unhealthy snack option (chocolate bar). Yet prior studies have demonstrated that nutritional labelling for unhealthy fast food does not encourage healthier purchasing (Elbel, Kersh, Brescoll, & Dixon, 2009), possibly because customers expect the food to be unhealthy. However, these studies only tested the effect of labelling, without testing for the relevance of the colour on nutritional labels. Consequently, it would be important to test the effects of colour labels on healthier snack options (e.g., cereal bar). Based on previous research in the fast food domain, one might predict a stronger effect of colour nutritional labels for healthier as compared to less healthy food options. Furthermore, effects of labels may be particularly potent for foods for which perceived and actual healthiness are discrepant, as in the case of cereal bars.

Moreover, the finding that a simple smiling or frowning emoticon can influence people's engagement with a certain type of behaviour may have far-reaching consequences for the design of very simple, engaging, easy to understand, and cost-effective population-based interventions that may have the added benefit of reaching the most deprived social groups. Importantly, combining smiling or frowning emoticons with different colours could make them even more potent signals of food healthiness. With these considerations in mind we examined the combined effects of emoticon expressions and colour nutritional labels, based on those used in Schuldt (2013). We aimed to test the relative impact of colour and emoticon labels on snack perceptions and choice, expecting that effects will not be additive in that emoticons will exert larger influence than colour due to their evolutionary significance in communicating important information between people (cf. Campos, Thein, & Owen, 2003).

## Methods

### Participants

The study was conducted amongst a nationally representative sample of 955 UK residents (47.1% female; *M_age_* = 50.31, *SD_age_* = 14.45) with sampling quotas set for SES occupational status (using the UK Registrar General's social classification; Pevalin & Rose, (2002): higher managerial and professional, white collar and skilled manual, and semi-skilled and unskilled manual). Our sample provided over 95% power at *α* = 0.05 to detect a medium-sized effect as reported by Schuldt (2013).

### Design

**Fig. 1:**
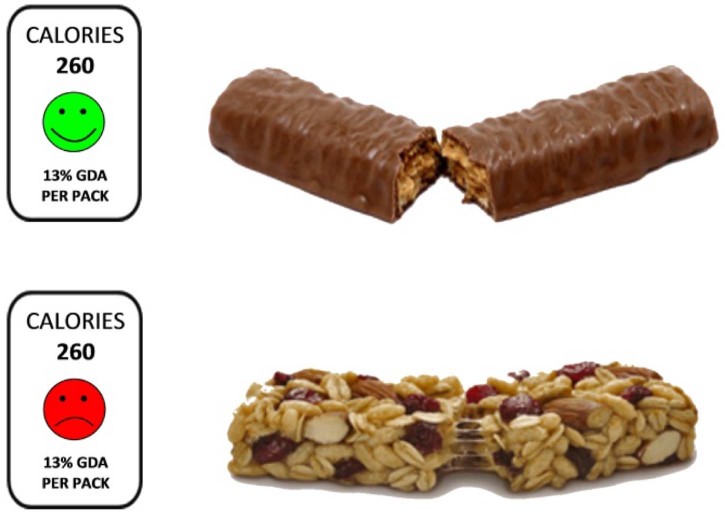
Sample of experimental stimuli.

The study had a 3 (emoticon expression: smiling vs. frowning vs. no emoticon) × 3 (colour label: red vs. green vs. white) × 2 (snack bar type: chocolate bar vs. cereal bar) between-subjects design. This gave 18 possible combinations of snack bar and label, one of which was randomly presented to each participant for the primary endpoint measures (for a sample see Fig. 1).

### Measures

#### Primary endpoints

Our primary endpoint measures were single-items adapted from Schuldt (2013), which target specific concrete constructs (e.g., desire to consume, see Bergkvist & Rossiter, 2007; Fuchs & Diamantopoulos, 2009).Current desire to consume the snack bar was measured by a single item: “*How much would you like to eat this chocolate bar (cereal bar) now?*” Responses were recorded on Likert-type scales anchored from *1* *=* *Not at all* to *9* *=* *Very much*.

Ratings of tastiness were measured by: “*Compared to other bars like this, how tasty is this chocolate bar (cereal bar)?*” Responses were given on Likert-type scales anchored from *1* *=* *Less tasty* to *9* *=* *More tasty*.

“*Compared to other bars like this, how healthy is this chocolate bar (cereal bar)?*” was an item used to assess healthiness ratings. Response options were anchored from *1* *=* *Less healthy* to *9* *=* *More healthy*.

Estimates of the calorific content were obtained by a single item: “*Compared to other bars like this, how many calories do you think this chocolate bar (cereal bar) contains?*” Answers were given on a scale from *1* *=* *Fewer calories* to *9* *=* *More calories*.

#### Secondary endpoint

After measuring the primary endpoint, participants were further randomised to see one of the nine combinations of chocolate bar and cereal bar shown in Table 5. We reduced the total number of possible combinations (full-factorial design: 81 pairs) to ease interpretation and retain statistical power. We opted against a fractional factorial design which would have included combinations with low face validity such as a label with a green frowning emoticon. The nine combinations selected were those that had the greatest face validity and, as such, had the highest likelihood of being implemented in practice. Participants saw one of the options and were asked to choose which they would prefer to eat right now.

#### Control variables

Hunger [*“How hungry do you feel right now?”*, answered on a 7-point rating scale anchored at −3 = *Very hungry, 0* *=* *Neither hungry nor full, +3* *=* *Very full*] and BMI were measured to examine whether there were any differences between participants randomised to different conditions that might affect the ratings of the snack foods.

### Procedure

The study was conducted online. The experiment was conducted in accordance with APA standards for the ethical treatment of human participants, and gained the prior approval by the Psychology Research Ethics Committee of the University of Cambridge. Electronic informed consent was obtained from all participants. In the first phase of the online study participants rated their current hunger levels. Then they were randomly assigned by the survey software platform Qualtrics to one of the 18 versions of the experiment. Once they completed the ratings on the primary endpoint, participants were asked to indicate their demographics, as well as weight and height, for BMI calculations. At the very end of the experiment, participants were further randomised into one of nine possible combinations of coloured emoticon labels and were asked to choose between a chocolate and a cereal bar (secondary endpoint).

## Results

### Randomisation checks

Two separate 3 (emoticon expression: smiling vs. frowning vs. no emoticon) × 3 (colour label: green vs. red vs. white) × 2 (snack bar type: chocolate bar vs. cereal bar) between participants' ANOVAs with hunger (all *Fs* < 1.1), and BMI (all *Fs* < 1.9) as dependent variables revealed no significant differences between the experimental conditions, indicating that randomisation of participants was successful. Furthermore, inclusion of hunger and BMI as covariates in the analyses did not affect the observed results for any of the dependent measures.

### Primary endpoints

#### Current desire to consume the snack bar

A 3 (emoticon expression: smiling vs. frowning vs. no emoticon) × 3 (colour label: green vs. red vs. white) × 2 (snack bar type: chocolate bar vs. cereal bar) between participants' ANOVA on desire to eat the given bar at that moment revealed a significant main effect of snack bar type, *F*(1, 937) = 47.51, *p* < .001, η^2^ = 0.047, whereby participants reported greater overall desire to eat the chocolate bar (*M* = 4.29, *SD* = 2.57) than the cereal bar (*M* = 3.23, *SD* = 2.23). For means and standard deviations see Table 1.

**Fig. 2:**
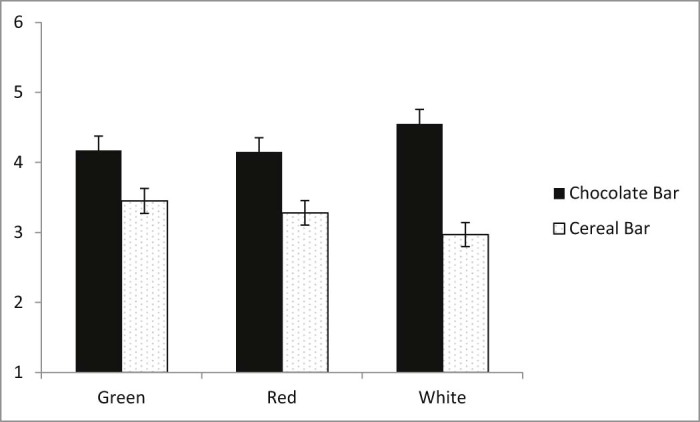
Significant interaction between snack bar type and colour label on current desire to consume the snack bar.

This effect was qualified by a marginally significant interaction between colour label and snack bar type, *F*(2, 937) = 2.71, *p* = .067, η^2^ = 0.005. An examination of simple main effects revealed that whilst overall the current desire for consumption was greater for the chocolate bar than the cereal bar across all three colour labels, this difference was most pronounced when the colour label was white, *F*(1, 937) = 33.98, *p* < .001, η^2^ = 0.035, and least pronounced when the colour label was green, *F*(1, 937) = 7.75, *p* = .005, η^2^ = 0.008, with red labels falling in the middle, *F*(1, 937) = 10.99, *p* = .001, η^2^ = 0.012 (see Fig. 2). No other effects were significant.

#### Ratings of tastiness

A 3 (emoticon expression: smiling vs. frowning vs. no emoticon) × 3 (colour label: green vs. red vs. white) × 2 (snack bar type: chocolate bar vs. cereal bar) between participants' ANOVA on tastiness ratings revealed a significant main effect of snack bar type, *F*(1, 937) = 8.06, *p* = .005, η^2^ = 0.008, whereby participants rated the chocolate bar (*M* = 5.11, *SD* = 1.55) as tastier overall than the cereal bar (*M* = 4.82, *SD* = 1.68). For a breakdown of means and standard deviations see Table 2.

The main effect was qualified by a significant three-way interaction, *F*(4, 937) = 2.86, *p* = .022, η^2^ = 0.012. Breaking down the three-way interaction revealed that there was a significant two-way interaction between food option and colour only when coupled with a frowning emoticon, *F*(2, 937) = 4.75, *p* = .009, η^2^ = 0.026, but not when coupled with a smiling emoticon or with no emoticon, *Fs* < 1.86, *ps* ≥ 0.156. An examination of simple main effects revealed that the largest difference in tastiness ratings between the chocolate bar (*M* = 5.44, *SD* = 1.62) and the cereal bar (*M* = 4.44, *SD* = 1.46) was obtained when there was a frowning emoticon expression on a white colour label, *F*(1, 937) = 10.59, *p* = .001, η^2^ = 0.011.

Furthermore, breaking down the three-way interaction differently revealed that there was a two-way interaction between food option and emoticon face expression when the label was white, *F*(2, 937) = 3.21, *p* = .040, η^2^ = 0.023, but not when the label was red or green, *Fs* < 2.28, *ps* ≥ 0.103. An examination of simple main effects showed that emoticon expression had a significant effect on ratings of tastiness for the cereal bar only when the colour label was white, *F*(2, 937) = 4.91, *p* = .008, η^2^ = 0.010, whereby tastiness ratings of the cereal bar were higher in the presence of a white smiling emoticon (*M* = 5.34) compared to a white frowning emoticon (*M* = 4.44) or no emoticon white label (*M* = 4.53). No other main effects or interactions were significant.

#### Ratings of healthiness

A 3 (emoticon expression: smiling vs. frowning vs. no emoticon) × 3 (colour label: green vs. red vs. white) × 2 (snack bar type: chocolate bar vs. cereal bar) between participants' ANOVA on healthiness ratings revealed a main effect of snack bar type, *F*(1, 937) = 33.38, *p* < .001, η^2^ = 0.033, whereby participants rated the cereal bar (*M* = 4.96, *SD* = 1.49) healthier than the chocolate bar (*M* = 4.43, *SD* = 1.31). There was also a main effect of emoticon expression, *F*(2, 937) = 3.20, *p* = .041, η^2^ = 0.006, whereby participants rated snack bars labelled with no emoticons as healthiest (*M* = 4.80, *SD* = 1.39), snack bars with smiling emoticons in the middle (*M* = 4.76, *SD* = 1.49), and snack bars with frowning emoticons as least healthy (*M* = 4.54, *SD* = 1.39). For a breakdown of means and standard deviations see Table 3.

**Fig. 3:**
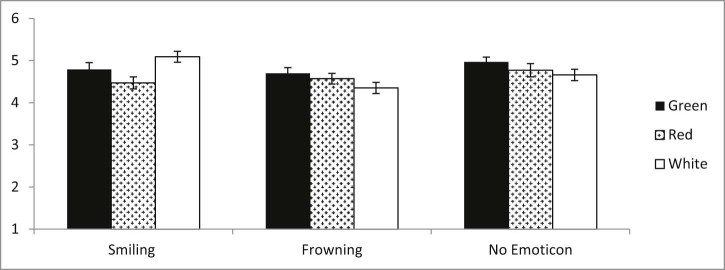
Significant interaction between emoticon expression and colour label on healthiness.

The main effects were qualified by a significant two-way interaction between colour label and emoticon expression, *F*(4, 937) = 2.80, *p* = .025, η^2^ = 0.011 (see Fig. 3). An examination of simple main effects revealed that smiling emoticons led to higher ratings of healthiness than frowning emoticons, but only when the colour label was white, *F*(2, 937) = 6.74, *p* = .001, η^2^ = 0.014, and not when the colour label was green or red, *Fs* < .98, *ps* ≥ 0.376. Breaking down the interaction differently, white colour labels led to higher healthiness ratings when compared to red and green colour labels only when they were accompanied by a smiling emoticon, *F*(2, 937) = 5.11, *p* = .006, η^2^ = 0.011, but not when they were accompanied by a frowning or no emoticon, *Fs* < 1.65, *ps* ≥ 0.193.

**Fig. 4:**
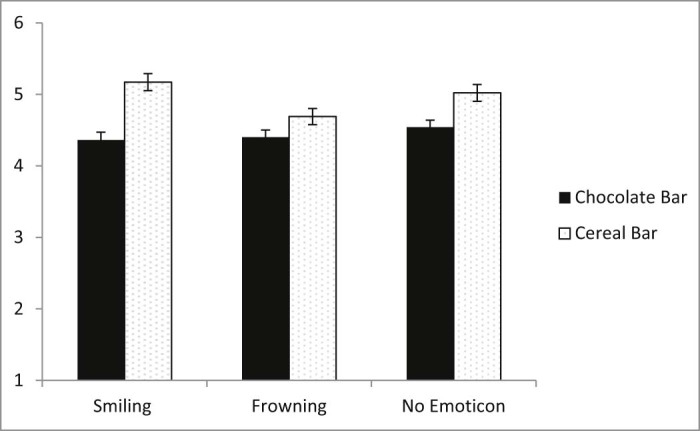
Significant interaction between snack bar type and emoticon expression on healthiness.

Furthermore, the effects were also qualified by a significant two-way interaction between emoticon expression and snack bar type, *F*(2, 937) = 2.99, *p* = .051, η^2^ = 0.006 (see Fig. 4). An examination of simple main effects revealed that the emoticon expression affected healthiness ratings of the cereal bar, but not ratings of the chocolate bar. Compared to neutral or smiling a frowning emoticon lowered ratings of healthiness for the cereal bar, *F*(2, 937) = 5.52, *p* = .004, η^2^ = 0.012, but did not lower healthiness ratings for the chocolate bar, *F* = .52, *ps* ≥ 0.595. No other main effects or interactions were significant.

#### Ratings of calories

A 3 (emoticon expression: smiling vs. frowning vs. no emoticon) × 3 (colour label: green vs. red vs. white) × 2 (snack bar type: chocolate bar vs. cereal bar) between participants' ANOVA on ratings of calories yielded no significant main effects or interactions, all *Fs* < .54. For means and standard deviations see Table 4.

### Secondary endpoint

A series of Chi-square tests were carried out to analyse the explicit choice of snack bar in the nine options that participants were randomised to see. The Chi-Square tests revealed that amongst all nine options participants chose the chocolate bar irrespective of the label (see Table 5).

We also carried out a binomial probability analysis whereby we tested the likelihood of choosing one of the snack bars whilst comparing the options where both snacks were equally encouraged (Options 1, 2 and 3) with those where chocolate choice was encouraged (Options 4, 6 and 9), and those where cereal bar choice was encouraged (Options 5, 7 and 8). The analysis yielded no significant results, all *ps* > .634.

## Discussion

In an experiment manipulating the colour and injunctive norm (emoticon expression) on nutritional labels, colour and emoticon expressions had mixed effects on perceptions and choice of snack foods. As predicted we did not find an additive effect on perceptions of labelled snack foods, in that emoticons, but not colour, affected perceptions of snacks. In particular, frowning emoticons on white background were most potent at modifying the ratings of tastiness and healthiness of snacks with a health halo (cereal bars). In line with recent systematic review evidence nutritional labels had no effect on choice of snacks. The efficacy of nutritional labelling therefore warrants further empirical investigation.

These findings extend the extant literature in several important ways. First, we failed to replicate Schuldt's (2013) findings that green labels increase the perception of healthiness of chocolate bars. However, our findings replicate prior studies that have found colour labels to be ineffective at guiding healthier food selection (e.g., Sacks et al., 2009, 2011). We also add to the recent systematic review of nutritional labelling which demonstrated the ineffectiveness of nutritional labels to increase the healthiness of food choices, as exemplified in our explicit choice task (see Crockett et al., under review). However, it is important to note that due to sampling constraints our explicit choice task (secondary endpoint) did not use a full-factorial design so it did not include labels with frowning emoticons on a white background, which may have been the most potent. However, frowning emoticons on white colour labels only decreased perceptions of healthiness and tastiness of cereal bars, and as chocolate bars tended to be chosen, these labels would be likely to sway choices towards less healthy options in the explicit choice task used in the present study. An explicit choice task thus would need to include another snack option that is both perceived-to-be and actually healthy in order to examine the effects of lowering already positive perceptions.

Our results also show that in the absence of colour there is an effect of emoticon labels, but there is no equivalent effect of colour in the absence of emoticons. One could perhaps argue that the non-significant effects of colour labelling are due to the fact that colour confuses people when emoticons are present. However, in our experimental design we also employed colour labels without emoticons as a control. Therefore, the absence of an effect of colour cannot be attributed to the presence of emoticons, since colour did not have an effect in the control conditions.

Finally, we show that injunctive norms, in the form of labels with simple emoticons on a white background may be more impactful at modifying consumers' healthiness perceptions than coloured labels. In particular, our results suggest that frowning emoticons may be more potent than smiling emoticons at signalling the healthiness and tastiness of food options, especially for foods that may have a health halo, such as cereal bars. These findings fit with prior theoretical and empirical work in psychology suggesting that bad/negative is stronger than good/positive (Baumeister, Bratslavsky, Finkenauer, & Vohs, 2001; Rozin & Royzman, 2001). These findings are also in line with prospect theory and the related loss aversion bias, whereby people are more sensitive to losses than to gains of the same magnitude (Kahneman & Tversky, 1979; Tversky & Kahneman, 1991). As such humans seem to be more attuned to bad or negative events and stimuli, and such stimuli elicit more thorough and elaborate information processing and learning (Fiske, 1980; Fiske & Taylor, 1991). Baumeister et al. (2001) proposed that this negativity bias is evolutionarily advantageous, since those individuals who are better attuned to negative stimuli would respond to threats more timely and adequately thus increasing their chances of survival and reproduction. Put simply, forgoing the possibility of a positive outcome may result in regret at a missed opportunity but nothing more, whilst failing to react to negative stimuli may be fatal instantaneously. Thus, the diagnostic value of negative and positive stimuli is asymmetrical in favour of negative information.

Of particular relevance to our findings, research by Öhman, Lundqvist, and Esteves (2001) demonstrated that negative affective expressions are better at capturing people's attention, by showing that schematically drawn threatening frowning faces are detected more quickly and accurately than happy smiling faces. In a similar vein, research suggests that people put in a positive mood use simple and intuitive solutions to problems, rely heavily on heuristics and use less information when making decisions (Isen & Means, 1983; Isen, Means, Patrick, & Nowicki, 1982). Conversely, inducing negative mood in people leads to deeper processing of information, more complex processing strategies, lower reliance on cognitive heuristics, and more systematic elaboration of complex messages (Bless, Bohner, Schwarz, & Strack, 1990; Fiedler, 1988; Isen et al., 1982; Sinclair, 1988). Taken together these diverse findings suggest that negative symbols, such as frowning emoticons, may be detected and processed quicker and deeper due to their diagnostic value to warn people of danger. Future research should further explore the proposed mechanisms underlying the effectiveness of frowning emoticon labels to discourage people away from unhealthy foods.

It is interesting to note that emoticon expressions had a significant effect on changing people's perception of the tastiness and healthiness of the cereal bar, but not the chocolate bar. These results are in line with prior research that has demonstrated that nutritional labelling in unhealthy fast food does not impact healthier purchasing (Elbel et al., 2009), possibly because consumers expect the food to be unhealthy. Furthermore, recent research has revealed that even though cereal bars are often high in sugar and fat content, many consumers have poor understanding of the unhealthiness of these snacks (Which, 2012). Thus, the diagnostic value of frowning emoticon labels may be greater for foods that possess a health halo, like cereal bars; whereas their diagnosticity may be lower for foods that people know are unhealthy, such as chocolate bars. In the context of this study, the value of the emoticon labels lies in the fact that they discourage rather than encourage people away from unhealthier foods that are incorrectly perceived as healthy.

Our findings of the effectiveness of injunctive norms in the form of emoticon expressions diverge from the findings by Robinson, Fleming, and Higgs (2014) who found that injunctive norms did not increase fruit and vegetable consumption in a student sample. However, an examination of their stimuli reveals that in their study injunctive norms were communicated in textual form, and there were no emoticon expressions to easily communicate norm approval/disapproval. These differences in the presentation of the injunctive norms as well as differences in our samples may have led to differences in results. Therefore, our results would suggest that to be effective injunctive norms need to be efficiently communicated to ease understanding, especially if aiming to reach a diverse sample. Moreover, their norms pertained to fruit and vegetable consumption amongst the student population. As such, they were trying to encourage the consumption of foods known to be healthy, rather than discourage people away from unhealthy snacks as in our study. Future studies should disentangle the differences between textual and pictorial presentations of injunctive norms, as well as the differential effectiveness of norm interventions to discourage vs. encourage people's consumption of different foods.

### Strengths and limitations of present research with suggestions for future work

A notable strength of our study is that we used a nationally representative sample of the UK population to examine the effects of colour and emoticon labelling on snack foods. Furthermore, we used an adequately powered sample to detect the effects of interest, so we could be more confident in drawing conclusions from our findings.

One of the limitations of our study is that we only measured self-reported preferences of the two snack options in an online study. Future studies should examine the effects of colour and emoticon labelling on more objective measures of behaviour, such as actual purchasing and consumption. Another limitation of our study is that for the primary endpoint we asked participants to rate their perceptions of a single product. Previous research has found that nutritional labels are more frequently used when comparing two different products, or when deciding about a product that is encountered for the first time (Malam et al., 2009). Further research may usefully extend the current findings in a within-subject design to emulate the decision processes in a supermarket where people can compare a multitude of different products before making a choice. Similarly, future research should test the effects of emoticon labels on selection of products within the same category as well as between foodstuffs, because there may be differential effects that would be important for implementation purposes. In a related vein future studies should examine these effects in real-world settings where food choices are made, such as supermarkets, restaurants, and canteens.

It is also important to note that we only examined the effects of labelling on snacks, therefore these effects may not be applicable to other types of food. Future research could usefully extend the present paradigm and examine the effects of emoticon labels on different types of foodstuffs, including main meals and sugar-sweetened beverages.

It is also plausible that the effects of the frowning emoticons only arise when the emoticons and the prior expectation of healthiness of the food option are discrepant. Thus, in our study a smiling emoticon label on a cereal bar matches participants' prior expectations, but a frowning emoticon does not. Future research should examine whether emoticon labels are especially potent for foods with discrepant perceived and actual healthiness. Such a study could usefully extend the present design to include food options that are perceived to be less healthy but are actually healthier, in order to ascertain whether the diagnostic value of the frowning emoticons arises from their greater potency as negative symbols, or from being coupled with unexpected/discrepant food options.

### Implications for future policy

Our results point to the need for further examination of the impact of colour labelling, especially in light of the growing popularity of traffic light labels. Policy decisions regarding traffic light and other similar colour labels should wait until the effects of such labels are systematically examined and the magnitude and direction of these effects are quantified.

Injunctive norms in the form of emoticon expressions may prove a beneficial addition to current nutritional labelling policies. Due to the universal nature of smiling and frowning (e.g., Ekman, 1972), and the communicative characteristic of such emotional expressions (Kraut & Johnston, 1979), using simple emoticon faces on labels of food should also make such interventions conducive for cross-cultural implementation. Population-level interventions utilising emotions and social norms may be particularly successful at guiding food choices due to the social aspects of eating behaviours in human societies. Moreover, emoticon labels on unhealthy foods may be more potent for children, who have been found to understand and act upon communicated emotions as early as infancy (Campos et al., 2003). As such, frowning emoticons may be more effective at signalling threat and danger arising from unhealthy food, since even very young infants can understand the non-verbal communicative significance of frowning and smiling emoticons, whilst the danger contained in the colour red, as used in traffic lights, may need to be learnt whilst growing up. This has important implications for devising policies to tackle the current childhood obesity epidemic (WHO, 2012).

### Conclusions

In keeping with recent systematic review evidence (Crockett et al., under review) overall labels had limited impact on perceptions, and no effects on choice of snacks. Emoticons on nutritional labels yielded stronger effects on perceptions of snacks than colour labels. Frowning emoticons in particular were more potent than smiling emoticons at signalling the healthiness and tastiness of cereal bars, which carry a health halo. The efficacy of colour nutritional labels requires further scrutiny. Population-level interventions aimed to encourage healthier food selection may benefit from communicating normative information.

## Figures and Tables

**Table 1:** Ratings of current desire to consume the snack bar as a function of emoticon expression and colour label.

	Emoticon expression					
	Smiling		Frowning		No emoticon	
Colour label	*M*	*SD*	*M*	*SD*	*M*	*SD*
	Chocolate bar					
Green	4.27	2.55	3.70	2.67	4.60	2.35
Red	4.14	2.69	4.20	2.59	4.11	2.44
White	4.46	2.39	4.61	2.82	4.57	2.51
	Cereal bar					
Green	3.19	2.13	3.25	2.30	3.84	2.27
Red	3.21	2.38	3.15	2.26	3.47	2.24
White	3.62	2.31	2.40	1.83	2.84	2.04

**Table 2:** Ratings of tastiness of snack bar type as a function of emoticon expression and colour label.

	Emoticon expression					
	Smiling		Frowning		No emoticon	
Colour label	*M*	*SD*	*M*	*SD*	*M*	*SD*
	Chocolate bar					
Green	5.22	1.70	4.72	1.85	5.21	1.28
Red	4.72	1.64	5.15	1.61	5.18	1.48
White	5.22	1.23	5.44	1.62	5.14	1.30
	Cereal bar					
Green	4.67	1.67	5.04	1.79	4.93	1.78
Red	5.02	1.64	4.60	1.63	4.75	1.65
White	5.34	1.73	4.44	1.46	4.53	1.69

**Table 3:** Ratings of healthiness of snack bar type as a function of emoticon expression and colour label.

	Emoticon expression					
	Smiling		Frowning		No emoticon	
Colour label	*M*	*SD*	*M*	*SD*	*M*	*SD*
	Chocolate bar					
Green	4.29	1.63	4.55	1.40	4.70	1.02
Red	4.14	1.34	4.39	1.17	4.48	1.46
White	4.72	1.07	4.28	1.43	4.43	1.04
	Cereal bar					
Green	5.37	1.29	4.84	1.41	5.19	1.22
Red	4.79	1.64	4.76	1.53	4.98	1.69
White	5.42	1.38	4.44	1.36	4.88	1.60

**Table 4:** Ratings of calories of snack bar type as a function of emoticon expression and colour label.

	Emoticon expression					
	Smiling		Frowning		No emoticon	
Colour label	*M*	*SD*	*M*	*SD*	*M*	*SD*
	Chocolate bar					
Green	5.69	1.70	5.51	1.64	5.47	1.33
Red	5.53	1.58	5.57	0.94	5.57	1.37
White	5.37	1.16	5.61	1.63	5.39	1.20
	Cereal bar					
Green	5.33	1.55	5.68	1.79	5.46	1.47
Red	5.45	1.62	5.51	1.79	5.58	1.50
White	5.58	1.32	5.70	1.56	5.47	1.75

**Table 5:** Total number and percentage of selections for the chocolate bar and cereal bar in each of the nine options respectively.

	Chocolate bar	Cereal bar	Chocolate bar *N (%)*	Cereal bar *N (%)*	Total *N*	*χ^2^*
Option 1			69 (69.7)	30 (30.3)	99	15.36***
Option 2			74 (67.9)	35 (32.1)	109	13.95***
Option 3			75 (64.1)	42 (35.9)	117	9.31**
Option 4			87 (74.4)	30 (25.6)	117	27.77***
Option 5			64 (66.7)	32 (33.3)	96	10.67***
Option 6			65 (65.7)	34 (34.3)	99	9.71**
Option 7			76 (68.5)	35 (31.5)	111	15.14***
Option 8			67 (60.9)	43 (39.1)	110	5.24*
Option 9			60 (61.9)	37 (38.1)	97	5.45*

*Note:* Significance denoted as *p ≤ .05; **p ≤ .01; ***p ≤ .001.
